# Vigilancia genómica de variantes del coronavirus del SARS-CoV-2 en pacientes hospitalizados: una cohorte del área metropolitana del valle de Aburrá, Colombia

**DOI:** 10.7705/biomedica.7783

**Published:** 2026-03-02

**Authors:** Olga H. Hernández-Ortiz, Laura Pérez-Restrepo, Jaime Úsuga, Melissa Moreno, Andrés Naranjo, Juan Vélez, Jorge Sará, Francisco Molina-Saldarriaga, Fabián Jaimes, Jorge Osorio, Juan P. Hernández-Ortiz

**Affiliations:** 1 Laboratorio Genómico One Health, Universidad Nacional de Colombia, Medellín, Colombia Universidad Nacional de Colombia Universidad Nacional de Colombia Medellín Colombia; 2 Cuidados Intensivos, Grupo Quirónsalud, Clínica Medellín, Medellín, Colombia Clínica Medellín Medellín Colombia; 3 Facultad de Medicina, Universidad de Antioquia, Medellín, Colombia Universidad de Antioquia Facultad de Medicina Universidad de Antioquia Medellín Colombia; 4 Cuidados Intensivos, Hospital General de Medellín, Medellín, Colombia Hospital General de Medellín Medellín Colombia; 5 Cuidados Intensivos, Grupo Auna, Clínica Las Américas, Medellín, Colombia Clínica Las Américas Medellín Colombia; 6 Palm Medical Center, Avon Park, Florida, United States. Avon Park Florida United States; 7 Clínica Universitaria Bolivariana, Universidad Pontificia Bolivariana, Medellín, Colombia Universidad Pontificia Bolivariana Universidad Pontificia Bolivariana Medellín Colombia; 8 Department of Pathobiological Sciences, School of Veterinary Medicine, University of Wisconsin-Madison, Madison, WI, United States University of Wisconsin University of Wisconsin-Madison Madison WI USA; 9 Facultad de Minas, Universidad Nacional de Colombia, Medellín, Colombia Universidad Nacional de Colombia Facultad de Minas Universidad Nacional de Colombia Medellín Colombia

**Keywords:** infecciones por coronavirus, proteína espícula del SARS-CoV-2, variantes del SARS-CoV-2, mutación del SARS-CoV-2, monitoreo epidemiológico, Coronavirus infections, spike protein, SARS-CoV-2, SARS-CoV-2 variants, SARS-CoV-2 mutation, epidemiological monitoring

## Abstract

**Introducción.:**

El coronavirus del SARS-CoV-2 ha sido una de las pandemias más significativas de la era moderna. Es un coronavirus con alta tasa de mutación, lo que genera variantes con cambios en la proteína espícula *(spike),* que dificulta su contención. La vigilancia genómica es crucial para adaptar estrategias de salud pública y vacunación, especialmente en los países de ingresos bajos y medios, donde el impacto ha sido considerable.

**Objetivo.:**

Identificar variantes virales y sus presentaciones clínicas en una cohorte prospectiva de pacientes hospitalizados con infección por SARS-CoV-2 entre octubre de 2021 y febrero de 2023.

**Materiales y métodos.:**

Se recopilaron datos demográficos, historial médico, estado de vacunación y progresión clínica. Se hizo secuenciación del genoma viral en muestras de hisopados nasales confirmadas con la prueba de reacción en cadena de la polimerasa en tiempo real con transcripción inversa para clasificar las cepas y detectar variantes.

**Resultados.:**

De 63 muestras positivas para SARS-CoV-2, la variante predominante fue ómicron (84 %), seguida de la delta (14 %). La variante delta presentó la mayor proporción de enfermedad grave (100 % casos críticos) comparada con ómicron (30 % casos críticos). Se identificaron mutaciones en la proteína espícula en cuatro categorías (A-D), junto con mutaciones específicas del gen S.

**Conclusión.:**

Los patrones de mutación observados coinciden con los reportes globales, con mayor seriedad clínica en la variante delta. Se identificaron mutaciones en la proteína espiga que confieren propiedades distintivas al virus. Estos hallazgos resaltan la necesidad de la vigilancia genómica continua para comprender la dinámica de la infección y orientar las estrategias de salud pública.

## Abstract (en)

**Introduction.:**

Severe acute respiratory syndrome coronavirus 2 (SARS-CoV-2) has been one of the most significant pandemics of the modern era. This coronavirus has a high mutation rate, resulting in variants with changes in the spike protein, which hinder containment efforts. Genomic surveillance is crucial for public health and vaccination strategies, especially in low-and middle-income countries, where the impact has been considerable.

**Objective.:**

To identify viral variants and their clinical presentations in a prospective cohort of hospitalized patients with SARS-CoV-2 infection between October 2021 and February 2023.

**Materials and methods.:**

We collected patients' demographic data, medical histories, vaccination status, and clinical progression. Viral genome sequencing was performed on nasopharyngeal swab samples confirmed by real-time reverse transcription polymerase chain reaction to classify strains and detect variants.

**Results.:**

The predominant variant in the 63 SARS-CoV-2-positive samples was omicron (84%), followed by delta (14%). A higher proportion of critical illness was observed in delta cases (100%) compared to omicron (30%). Mutations in the spike protein were identified and classified into four categories (A-D), along with specific mutations in the *S* gene.

**Conclusion.:**

The mutational patterns observed in this study are consistent with global reports, with delta cases presenting greater clinical severity. We identified spike protein mutations that may confer distinctive properties to the virus. These findings underscore the need for ongoing genomic surveillance to better understand viral dynamics and guide public health strategies.

El coronavirus del SARS-CoV-2 *(Severe Acute Respiratory Syndrome Coronavirus 2)* es el agente causal de la enfermedad por COVID-19 *(Coronavirus Disease* 2019) que desencadenó una de las pandemias más significativas de la era moderna, y afectó a millones de personas en todo el mundo desde su aparición a finales del 2019. La rápida diseminación del virus y su capacidad para causar enfermedad grave, especialmente en individuos vulnerables, dio lugar a una crisis de salud pública sin precedentes ^1^.

El SARS-CoV-2 es un virus de ácido ribonucleico (ARN) de cadena sencilla y polaridad positiva, perteneciente a la familia de los coronavirus. La ausencia de actividad de corrección de errores en su polimerasa viral favorece una alta frecuencia de mutaciones y la generación de múltiples variantes ^2^^,^^3^.

Esta capacidad de mutación, común entre los virus de ARN, permite que el SARS-CoV-2 adquiera una notable diversidad genética a medida que se replica en la población ^4^. Las variantes resultantes pueden presentar alteraciones significativas en la estructura de sus proteínas, especialmente en la proteína de la espícula *(spike),* la cual es fundamental para la entrada del virus en las células humanas. A lo largo de la pandemia, la evolución del SARS-CoV-2 dio lugar a múltiples variantes con un impacto considerable en la dinámica de transmisión y en los esfuerzos de control ^5^. Las tasas de transmisión y mortalidad variaron sustancialmente entre regiones, influenciadas por factores como las políticas sanitarias, la aparición de nuevas variantes y el acceso a la vacunación ^1^^,^^5^.

En este contexto, la nomenclatura y la clasificación de las variantes ha sido clave para su vigilancia y estudio. En mayo del 2021, la Organización Mundial de la Salud (OMS) introdujo un sistema de denominación basado en las letras del alfabeto griego, diseñado para facilitar la comunicación pública y evitar la estigmatización de los países donde fueron identificadas por primera vez ^6^.

Por otra parte, el sistema de *software* Linajes Pango implementado desde abril del 2020, permite el rastreo filogenético del virus mediante la identificación de linajes como BA.1 o BA.2, a partir de los datos de la secuenciación genómica ^7^.

Además, la herramienta bioinformática Nextclade emplea nomenclaturas como 21L o 20B y clasifica los virus en clados definidos por conjuntos específicos de mutaciones y sus relaciones filogenéticas, contribuyendo al seguimiento detallado de la evolución viral ^8^.

Entre las variantes más notables, según la clasificación de la OMS, por ser variantes de preocupación, se encuentran: alfa, beta, gamma, delta y, posteriormente, ómicron. Cada una de ellas presentaba mutaciones que afectaron la estructura de la proteína de la espícula.

Estas variantes surgieron en distintas regiones del mundo y se diseminaron con rapidez, favorecidas por la alta transmisibilidad del virus y el movimiento de la población globalmente ^6^. En particular, ómicron -detectada por primera vez en Sudáfrica a finales del 2021- puso en evidencia la velocidad con la que una variante con mayor capacidad de evasión del sistema inmunológico puede expandirse a nivel mundial ^9^.

En Colombia, la circulación de variantes del SARS-CoV-2 reflejó en gran medida, por un lado, las tendencias observadas a nivel global y, por otro, las características particulares de cada zona, determinadas por las capacidades del sistema de vigilancia epidemiológica y la heterogeneidad geográfica de la población. Variantes como la gamma (originada en Brasil) y la delta (detectada inicialmente en India) ingresaron al país en distintos momentos y estuvieron asociadas con olas epidémicas sucesivas ^10^.

Un caso particular fue el de la variante mi, detectada por primera vez en Colombia en enero del 2021, la cual alcanzó una elevada prevalencia, que representó cerca del 50 % de los casos de COVID-19 durante el tercer pico epidémico ^11^^,^^12^. Esta variante presentó un perfil mutacional distintivo, particularmente en la proteína de la espícula, lo que incrementó su capacidad de evasión frente a la neutralización por anticuerpos ^13^^,^^14^.

Posteriormente, la variante ómicron se convirtió en la dominante en el país, con un acentuado incremento de casos a inicios del 2022 ^15^.

Las mutaciones presentes en las distintas variantes han representado desafíos constantes para la eficacia de las vacunas y la implementación de medidas de control en Colombia ^10^. Dada la amplia diversidad de variantes y linajes del SARS-CoV-2, resulta difícil predecir con exactitud el comportamiento clínico asociado a cada uno de ellos ^16^^-^^18^. Así pues, no se puede asegurar la capacidad protectora de las vacunas frente a futuras variantes, debido a la dinámica cambiante de las mutaciones virales ^19^^,^^20^. En consecuencia, es fundamental fortalecer la vigilancia genómica a nivel nacional, con el fin de monitorear la aparición de nuevas variantes y adaptar oportunamente estrategias de salud pública ^16^^-^^18^.

En este contexto, el presente estudio tuvo como objetivo hacer vigilancia genómica de las variantes circulantes en una cohorte del área metropolitana del valle de Aburrá, durante el periodo entre octubre del 2021 y febrero del 2023. Este tipo de vigilancia constituye una herramienta clave para la toma de decisiones clínicas, preventivas y terapéuticas, ya que permite ajustar las estrategias de vacunación, guiar el desarrollo de nuevas vacunas y comprender en profundidad la dinámica genética y de propagación del virus y de sus variantes.

## Materiales y métodos

### Diseño del estudio

Se realizó un estudio prospectivo de una cohorte de pacientes hospitalizados en salas generales o en unidades de cuidados intermedios o intensivos entre octubre del 2021 y febrero del 2023 en centros clínicos y hospitalarios ubicados en municipios del área metropolitana del valle de Aburrá.

### Criterios de inclusión y exclusión

Los criterios de elegibilidad fueron: pacientes de 18 años o más, con infección aguda por SARS-CoV-2, hospitalizados en salas generales o en unidades de cuidados especiales o intensivos entre octubre del 2021 y febrero del 2023, en uno de los siguientes centros hospitalarios: Hospital General de Medellín; Clínica Las Américas-Auna; Hospital Pablo Tobón Uribe; Hospital Universitario San Vicente Fundación; Clínica Alma Máter de Antioquia; Clínica Medellín-Grupo Quirónsalud; Clínica CES o Clínica Universidad Bolivariana, ubicadas en municipios del área metropolitana del valle de Aburrá (Bello, Medellín, Itagüí, Envigado, Sabaneta, La Estrella, Caldas, Barbosa, Copacabana y Girardot).

Se incluyeron pacientes cuya secuenciación del genoma viral presentó una cobertura mínima del 40 % y que, además, contaban con confirmación previa de su linaje mediante la prueba de reacción en cadena de la polimerasa en tiempo real con transcripción inversa. Los pacientes con una orden de no reanimación y aquellos que no contaban con un número de teléfono para hacer el seguimiento fueron excluidos de este estudio.

### Datos y recolección de la información

Se diseñó un formulario de encuesta clínico-epidemiológica para los participantes (anexo 1) en el que se registró:

1. información demográfica;
2. historial médico, incluido el uso de medicamentos;
3. antecedentes de infecciones por COVID-19 y vacunación;
4. progresión de los síntomas;
5. afecciones médicas como hipertensión, diabetes mellitus, enfermedad pulmonar obstructiva crónica, enfermedad renal u obesidad, entre otras;
6. antecedentes de inmunosupresión, incluida quimioterapia, terapias biológicas y enfermedades autoinmunitarias, y
7. antecedentes socioeconómicos.

Los médicos diligenciaron esta encuesta con los datos correspondientes en el momento del ingreso al estudio.

Se hizo seguimiento de la infección aguda de los pacientes hospitalizados, registrando la información sobre la duración de la estancia hospitalaria, el traslado a la unidad de cuidados intensivos o especiales y la clasificación de la enfermedad según su gravedad.

Se llevaron a cabo evaluaciones clínicas interdiarias diseñadas por los investigadores, que tenían como base los datos del seguimiento cotidiano por los médicos tratantes de los pacientes. Además, se monitorearon las fechas de egreso, registrando el estado vital en el momento del alta hospitalaria y la mortalidad a los 28 días de la infección por SARS-CoV-2. Se registró la información sobre los días de respiración mecánica asistida, diálisis o choque. Luego, se hizo seguimiento a los participantes por medio de llamada telefónica en el día 28 después de la infección para completar el registro de datos.

### Vigilancia genómica para SARS-CoV-2

**Figura 1:**
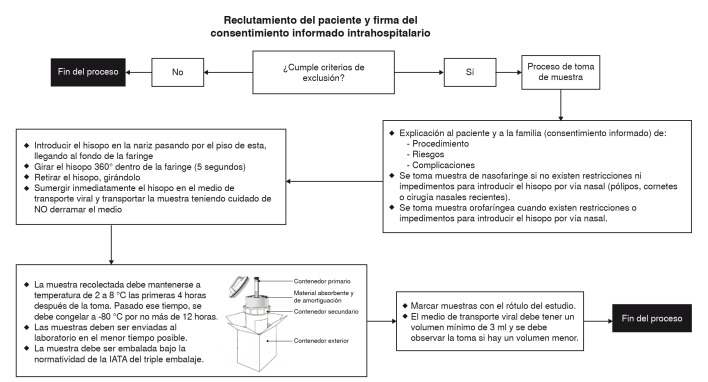
Flujograma de hisopado nasofaríngeo u orofaríngeo.

En las primeras 72 horas después del ingreso del paciente al estudio en los centros participantes, se tomó una muestra de hisopado nasal para practicar una prueba de reacción en cadena de la polimerasa en tiempo real con transcripción inversa siguiendo el proceso que se muestra en la figura 1. Esta prueba se llevó a cabo en un termociclador CFX96™ de Laboratorios Bio-Rad utilizando el kit iTaq™ *Universal Probes One-Step* de Biorad y cebadores o sondas específicas dirigidas a los genes de la envoltura y de la ARN polimerasa dependiente de ARN (cuadro 1).

**Cuadro 1:** Cebadores y sondas

Cebadores	Secuencia de cebadores y sondas
E Sarbeco Forward	ACAGGTACGTTAATAGTTAATAGCGT
E Sarbeco Reverse	ATATTGCAGCAGTACGCACACA
E Sarbeco Probe	**FAM**-ACACTAGCCATCCTTACTGCGCTTCG-BHQ-1
RNasaP Forward	AGATTTGGACCTGCGAGCG
RNasaP Reverse	GAGCGGCTGTCTCCACAAGT
RNasaP Probe	**Cy5**-TTCTGACCTGAAGGCTCTGCGCG-**BHQ-1**
RdRP Forward	GTGARATGGTCATGTGTGGCGG
RdRP Reverse	CARATGTTAAASACACTATTAGCATA
RdRP Probe P1	**FAM**-CCAGGTGGWACRTCATCMGGTGATGC-**BBQ**
RdRP Probe P2	**FAM**-CAGGTGGAACCTCATCAGGAGATGC-**BBQ**

BBQ: *blackberry quencher*; BHQ: *black hole quencher*; E: gen E; FAM: 6-carboxifluoresceína; RdRP: ARN polimerasa dependiente de ARN; RNasaP: ribonucleasa P.

Las condiciones de ejecución fueron: un ciclo a 50 °C durante 10 min, un ciclo a 95 °C durante 3 min y 40 ciclos de 95 °C durante 15 s y 58 °C durante 30 s. Además, para cada muestra se realizó una amplificación del gen de la ribonucleasa P como control de la extracción del ARN viral.

Luego, se llevó a cabo un cálculo del ciclo umbral para cada una de las muestras según las curvas de la prueba de reacción en cadena. El valor obtenido corresponde al ciclo en el que la señal de fluorescencia supera una unidad relativa de 300 para el gen *E* y el ARN polimerasa dependiente de ARN, y de 100 para el gen de la ribonucleasa P. Las muestras se consideraron positivas cuando el valor del ciclo umbral fue inferior a 38. La extracción del ARN se realizó con una columna de sílice utilizando el kit de extracción viral de Zymo Research™, siguiendo las instrucciones del fabricante y el protocolo descrito por Corman *et al.* y por los *National Institutes of Health* (NIH) para la detección del gen *E* y del ARN polimerasa dependiente de ARN ^21^.

*Secuenciación del genoma completo:* las muestras con un valor del ciclo umbral inferior a 27 fueron seleccionadas para la secuenciación el protocolo nCoV-2019, versión 4.1 ^22^. Primero, se sintetizó el ARN complementario a partir de 8 μl utilizando el kit LunaScript RT Supermix™ (5X). Posteriormente, se realizó una prueba de reacción en cadena de la polimerasa multiplex empleando 218 cebadores distribuidos en dos grupos (grupo 1: 110 cebadores; grupo 2: 108 cebadores) y utilizando la mezcla maestra Q5 Hot Start High-Fidelity™ 2X de New England Biolabs, Estados Unidos. Para cada muestra se realizaron dos reacciones de amplificación independientes a partir de 3 μl de ADN complementario.

Los productos de la PCR se agruparon mezclando 5 μl de cada reacción con 40 μl de agua libre de nucleasas. Cada mezcla de amplicones fue cuantificada mediante el kit Qubit dsDNA HS Assay™ de Thermo Fisher Scientific. Las mezclas con concentraciones iguales o superiores a 7 ng/μl se seleccionaron para la etapa de reparación, la cual se realizó con el NEBNext Ultra™ II End Repair/dA-Tailing Module de New England Biolabs y con una cantidad de entre 50 y 100 ng de ADN.

La ligadura del código de barras se efectuó utilizando el kit Native Barcoding Expansion 96™ de Oxford Nanopore, junto con la Blunt/TA Ligase Master Mix™ de New England Biolabs. Posteriormente, las muestras fueron agrupadas y purificadas con las perlas AMPure XP™ de Beckman coulter.

La ligadura del Adapter Mix II™ de Oxford Nanopore Technologies se llevó a cabo con el kit Quick Ligation™ de New England Biolabs, seguida de una segunda purificación con las perlas AMPure XP. Finalmente, el ADN purificado se mezcló con tampón Sequencing Buffer™ y con perlas de carga y se almacenó en la celda de flujo R9 para su secuenciación.

El ensamblaje de los datos brutos de secuenciación de nueva generación se realizó siguiendo el flujo de análisis bioinformático *(pipeline)* descrito en la plataforma de Oxford Nanopore Technologies ^22^. Cada una de las secuencias obtenidas fue analizada mediante la herramienta Pango-Lineage, que permite asignar un linaje con base los reportes hasta la fecha ^23^. Se utilizó Nextclade para identificar y caracterizar las mutaciones presentes en cada secuencia.

*Análisis filogenético:* para la construcción del árbol se descargaron, aproximadamente, 5000 secuencias completas del genoma del SARS-CoV-2 desde la plataforma GISAID *(Global Initiative on Sharing All Influenza Data).* Se siguió el protocolo descrito por Jolly y Scaria ^24^ para los análisis filogenéticos del SARS-CoV-2, empleando el paquete Augur, versión 24.1.0, de Nextstrain ^25^. Las secuencias fueron alineadas contra la referencia MN908947.3 utilizando *Augur align* y, posteriormente, se construyó el árbol filogenético mediante IQTREE™, versión 1.6.1 ^26^.

La resolución temporal de la filogenia se obtuvo con *Augur Refine* y la asignación de clados se realizó con *Augur Clades,* que incorpora las inferencias de cambios nucleotídicos y aminoacídicos generadas por *Augur Ancestral* y *Augur Translate,* respectivamente. Por último, el árbol resultante fue visualizado mediante el servidor Auspice, siguiendo la clasificación de clados definidos por Nextclade.

### Clasificación de los efectos biológicos

#### Identificación de los cambios de aminoácidos

Las secuencias de estudio fueron alineadas utilizando el programa MAFFT™ *(Multiple Alignment with Fast Fourier Transform, version 7)*^27^. Posteriormente, se recortó el segmento correspondiente a la proteína S, con base en la anotación del gen *S* de la secuencia de referencia hCoV-19/ Wuhan/WIV04/2019, utilizando AliView™, v1.0 ^28^.

Se desarrolló un código en Python para comparar las secuencias de aminoácidos de las muestras con la secuencia de referencia y se hizo el conteo de cambios de aminoácidos por posición para cada variante. Luego, se generó un gráfico combinado que muestra la frecuencia de cambios de aminoácidos a lo largo de cada posición de la proteína S, utilizando la librería de Python dna_features_viewer ^29^. Finalmente, se pintaron los dominios de la proteína utilizando Inkscape™, versión 1.3.2.

#### Clasificación de los cambios de aminoácidos

Para la identificación de mutaciones se utilizó la aplicación CovSurver *(Coronavirus Surveillance),* disponible en la plataforma GISAID, empleando como referencia la cepa hCoV-19/Wuhan/WIV04/2019. Esta herramienta permitió, además, clasificar cada uno de los cambios de aminoácidos de acuerdo con su efecto biológico (cuadro 2) y su relevancia epidemiológica ^30^. Para los análisis, se tuvieron en cuenta los cambios de aminoácidos que están presentes, por lo menos, en el 25 % de las muestras que presentaron cambios que tiene efectos fenotipos registrados en la literatura (cuadro 3).

**Cuadro 2:** Clasificación de los efectos biológicos obtenidos de la aplicación *Coronavirus Surveillance* (CovSurver).

Categoría	Efecto biológico
A	Ningún efecto biológico
B	Sustituciones en sitios con posibles efectos fenotípicos
C	Implicaciones en la unión al receptor de la célula huésped o la antigenicidad
D	Eliminación de algún sitio de glicosilación

**Cuadro 3:** Descripción de mutaciones de la COVID-19.

Mutaciones	Implicaciones biológicas	Fuente: GISAID	Ocurrencia GISAID (%)
S: T19I	T19I es un cambio de aminoácido en la proteína de pico de los genomas del SARS-CoV-2, que es específica de la variante BA.2 (ómicron). Este cambio se relaciona con la eliminación de un sitio de N-glicosilación en la posición 17 que puede afectar las propiedades antigénicas.	https://pubmed.ncbi.nlm.nih.gov/36985302/	32,32
S: G142D	G142D es un cambio de aminoácido que ocurre en el dominio NTD de la proteína S del SARS-CoV-2. Se han reportado cambios en las propiedades antigénicas, donde la presencia de este cambio confiere resistencia al mAb COV2-2489.	https://pubmed.ncbi.nlm.nih.gov/36948931/	85,15
S: G339D	G339D es un cambio en el dominio RBD de la espiga, reportado en el linaje ómicron. Este cambio se puede relacionar con cambios en la afinidad de unión a ACE2.	https://pubmed.ncbi.nlm.nih.gov/34033342/ https://www.ncbi.nlm.nih.gov/pmc/articles/PMC7310626/	73,64
S: S371F	S371F es conjunto con las mutaciones D405N y R408S, que se encuentran en el linaje ómicron y estas socavan la mayoría de los anticuerpos neutralizantes de sarbecovirus, debido a que estas mutaciones inducen cambios conformacionales en el bucle 366-377.	https://www.ncbi.nlm.nih.gov/pmc/articles/PMC9385493/	31,33
S: S373P	S373P es un cambio de aminoacidos relacionado con el aumento de afinidad de unión con la ACE2 humano, por ello se dice que disminuyen la estabilidad de las proteínas, pero aumentan los riesgos de enfermedad.	https://pubmed.ncbi.nlm.nih.gov/34033342/ https://onlinelibrary.wiley.com/doi/10.1002/jmv.27526	74,57
S: S375F	El cambio S375F se relaciona con un aumento de la afinidad por la unión con la ACE2 humano.	https://pubmed.ncbi.nlm.nih.gov/34914115/	74,36
S: T376A	El cambio T376A en el dominio de unión al receptor ACE2, en sinergia con otros cambios afectan gravemente la infección mediada por S, al afectar el procesamiento de S, aunque no están ubicados cerca del sitio de escisión de furina S1/S2.	https://www.ncbi.nlm.nih.gov/pmc/articles/PMC9289044/	31,45
S: D405N	El cambio D405N inhibe la infección por ómicron BA.5 de HL-mEC (células endoteliales microvasculares primarias de pulmón humano, ACE2-negativas.), debido a la falta de afinidad de Spike por las integrinas.	https://www.ncbi.nlm.nih.gov/pmc/articles/PMC9962894/	32,17
S: R408S	El cambio R408S está implicado en cambios conformacionales de la proteína que afectan la unión de anticuerpos de la cadena pesada de los anticuerpos NAbs del grupo F3 neutralizantes.	https://www.ncbi.nlm.nih.gov/pmc/articles/PMC9385493/	30,92
S: K417N	El cambio K417N contribuye al escape inmunológico de los anticuerpos neutralizantes en los grupos A-D, cuyos epítopos se superponen con el motivo de unión a ACE2. debido a una interacción crítica de puente salino entre Lys417 y residuos cargados negativamente en los anticuerpos.	https://www.ncbi.nlm.nih.gov/pmc/articles/PMC8866119/	66,77
S: N440K	N440K es un cambio con implicaciones en el escape de la neutralización de anticuerpos.	https://pubmed.ncbi.nlm.nih.gov/34904526/	66,61
S: L452R	El cambio L452R, junto con F490 y L492, forman un parche hidrofóbico en la superficie del dominio RBD, lo que estabiliza la interacción entre la proteína de pico y su receptor ACE2 humano y así aumentar la infectividad.	https://pubmed.ncbi.nlm.nih.gov/33991487/	15,60
S: S477N El	El cambio S477N en conjunto con otros cambios muestran una mayor resistencia a la neutralización por sueros vacunados.	https://www.ncbi.nlm.nih.gov/pmc/articles/PMC9877909/	76,02
S: T478K	El cambio de aminoácido T478K se relaciona con una modificación electrostática en la superficie de la proteína al sustituir un aminoácido no cargado (treonina) por uno positivo (lisina), que afecta la interacción con ACE2	https://www.ncbi.nlm.nih.gov/pmc/articles/PMC8242375/	88,01
S: E484A	El cambio E848A causa una pérdida de aptitud física debido a que se ha asociado con una interfaz de unión de ACE2 más pequeña, lo que aboga por una disminución de la afinidad de ACE2	https://www.ncbi.nlm.nih.gov/pmc/articles/PMC10633978/	73,83
S: F486V	El cambio F486V permite escapar de este grupo de potentes anticuerpos neutralizantes.	https://www.ncbi.nlm.nih.gov/pmc/articles/PMC10349087/	3,26
S: Q498R	El cambio Q498R, junto con N501Y, S371L, S373P, S375F, Q493R y T478K contribuyen significativamente a una alta afinidad de unión con la ACE2 humana	https://onlinelibrary.wiley.com/doi/10.1002/jmv.27526	74,08
S: N501Y	El cambio N501Y mejora la estabilidad de la proteína de pico y contribuyen significativamente a una alta afinidad de unión con la ACE2 humana.	https://onlinelibrary.wiley.com/doi/10.1002/jmv.27526	76,46
S: Y505H	Es un cambio comúnmente observado en las variantes delta y ómicron. Se encuentra en el bucle Q498-Y505 dentro de RBD, que es fundamental para la interacción de RBD y ACE2. Este cambio en la disminución de la estabilidad de la proteína.	https://www.ncbi.nlm.nih.gov/pmc/articles/PMC10056712/	73,92
S: D614G	G614G se asocia con una mayor infectividad, así como evidencia clínica de que posee cargas virales más altas. Está ubicada en la superficie del protómero de la proteína Spike, donde puede formar contactos con el protómero vecino mediante un enlace de hidrógeno. El cambio de aminoácido elimina el enlace de hidrógeno y aumentará la flexibilidad en la interacción entre protómeros.	https://pubmed.ncbi.nlm.nih.gov/32697968/	99,34
S:H655Y	H655Y aumenta la infectividad del SARS-CoV-2 al fortalecer la afinidad por ACE2. El cambio promueve una conformación RBD más abierta, en el camino para la unión de ACE2. Por otro lado, las interacciones introducidas por H655Y es una interacción polar con T696. Esta interacción une las cadenas polipeptídicas S1 y S2 y estabilizará el dominio C-terminal, haciéndolo menos propenso a la disociación después del procesamiento proteolítico en el sitio de escisión S1/S2.	https://pubmed.ncbi.nlm.nih.gov/37034621/	82,43
S: N679K	El cambio N679K junto con P681H, presentes en ómicron, promueven la aparición de un nuevo sitio de escisión para la catepsina G, lo que posiblemente podría aumentar la infectividad y la transmisibilidad.	https://www.ncbi.nlm.nih.gov/pmc/articles/PMC9015119/	82,07
S: P681H	El cambio P681H puede aumentar ligeramente la escisión S1/S2, pero esto no afecta significativamente la entrada viral o la propagación entre células. P681H reduce la dependencia de las catepsinas endosómicas, lo que coincide con una mayor entrada a la superficie celular. Por otro lado, este cambio de aminoácido juega un papel fundamental en la resistencia al interferón IFN-β en las células epiteliales del pulmón humano.	https://www.ncbi.nlm.nih.gov/pmc/articles/PMC8043443/ https://www.ncbi.nlm.nih.gov/pmc/articles/PMC9749455/	81,69
S: N764K	Los cambios de aminoacidos T547K, H655Y, N764K, N856K, N969K, L981F en la variante ómicron mejoran en gran medida la interacción S2:ED1 (el dominio de encapsulación 1), compensando parcialmente la pérdida de la interacción S2:ED2 debido a la mutación D614G.	https://www.ncbi.nlm.nih.gov/pmc/articles/PMC9638846/	77,18
S: D796Y	Es un cambio de aminoácido común en la proteína S del linaje ómicron.	https://pubmed.ncbi.nlm.nih.gov/34033342/	80,70
S: Q954H	Se cree que este cambio podría alterar los epítopos estructurales importantes, que son objetivos de los mAb terapéuticos en aplicaciones clínicas.	https://www.ncbi.nlm.nih.gov/pmc/articles/PMC10389082/	78,61
S: N969K	N969K por sí sola es suficiente para inducir un desplazamiento sustancial en la estructura de la columna vertebral de repetición de heptada 2 (HR2) en el paquete de posfusión HR1HR2. Debido a esta mutación, los inhibidores de péptidos de entrada de fusión basados en la secuencia de la cepa de Wuhan son menos eficaces.	https://www.ncbi.nlm.nih.gov/pmc/articles/PMC10068829/	78,72

ACE2: enzima convertidora de angiotensina 2; BA.2: sublinaje BA.2 de la variante ómicron; D: ácido aspártico; ED1: dominio de encapsulación 1; ED2: dominio de encapsulación 2; GISAID: *Global Initiative on Sharing All Influenza Data;* HL-mEC: células endoteliales microvasculares primarias de pulmón humano; HR1: repetición de heptada 1; HR1HR2: complejo de repetición de heptada 1 y 2; HR2: repetición de heptada 2; IFN-ß: interferón beta; mAb: anticuerpo monoclonal; NAbs: anticuerpos neutralizantes; NTD: dominio N-terminal; RBD: dominio de unión al receptor; S: proteína de la espícula; S1/S2: sitio de escisión entre las subunidades S1 y S2; S2: subunidad 2 de la proteína de la espícula; SARS-CoV-2: *Severe acute respiratory syndrome coronavirus 2.*

#### Posición de los cambios en la proteína

Para identificar la posición espacial de los cambios de aminoácidos, se descargó la estructura del trímero de la proteína espícula en estado cerrado. La estructura tridimensional se obtuvo del *Protein Data Bank,* bajo el código 7DDD ^31^. Las proteínas fueron editadas utilizando PyMOL, versión 3.0.3 (Python Molecular Graphics System, Schrödinger).

Se resaltaron las posiciones en las que se presentaron cambios de aminoácidos en las secuencias de estudio en comparación con la secuencia de referencia (NC_045512). Los residuos se colorearon según la frecuencia del cambio observada en las secuencias analizadas. Asimismo, se señalaron las posiciones correspondientes a los dominios *Receptor Binding* y *N-Terminal,* utilizando Inkscape, versión 1.3.2.

### Variables

• Clasificación de la variante viral

» Esquema de la OMS de letras griegas 

» Clasificación de linajes Pango 

» Sistema NextClade

• Clasificación clínica de la infección aguda por SARS-CoV-2

» Enfermedad moderada: manifestaciones clínicas de neumonía (fiebre tos, disnea y taquipnea), pero con saturaciones de oxígeno mayores a 94 % al aire ambiente.

» Enfermedad grave: neumonía sumada a frecuencia respiratoria mayor de 30 respiraciones por minuto; evidencia de dificultad respiratoria o de saturación de oxígeno (menor 94 %) al aire ambiente; y relación entre la presión arterial de oxígeno y la fracción inspirada de oxígeno menor a 300 mm Hg; en imágenes, opacidades pulmonares que comprometían más del 50 % del parénquima pulmonar en las primeras 24 a 48 horas.

» Enfermedad crítica: presencia de choque séptico, compromiso cardiaco, falla respiratoria o síndrome de disfunción multiorgánica.

- Soporte con respiración mecánica asistida, diálisis o choque
- Edad
- Sexo

### Control de sesgos

Para controlar los sesgos de información y selección, la investigadora principal auditó los registros para minimizar errores en la recolección de datos. Ante valores extremos o inconsistentes, se revisaron nuevamente las historias clínicas. Además, el personal del estudio fue capacitado y los instrumentos se estandarizaron antes de su aplicación, reduciendo el sesgo del entrevistador.

Para evitar pérdidas de seguimiento y controlar el sesgo correspondiente, se utilizaron los datos disponibles de la cohorte de pacientes. Además, se recolectaron datos de contacto, lo cual permitió confirmar información importante y reducir el sesgo de memoria.

Las muestras biológicas fueron procesadas en el Laboratorio Genómico *One Health* de la Universidad Nacional, sede Medellín, el cual opera bajo condiciones de bioseguridad de nivel 2 y cumple con los estándares de calidad requeridos, lo que permitió minimizar el sesgo de medición.

### Estadística aplicada

Se aplicó estadística descriptiva para las variables cuantitativas mediante medidas de tendencia central y dispersión (media, desviación estándar, mediana, cuartiles y rango), de acuerdo con la distribución de los datos (normal o no). Las variables cualitativas se presentaron mediante tablas de frecuencia y porcentajes sobre el total de respuestas evaluables.

Las comparaciones de variables categóricas entre variantes se realizaron mediante la prueba de c^2^ o la prueba exacta de Fisher. Para las variables continuas con distribución normal, se utilizó la prueba t de Student, mientras que para aquellas con distribución no normal se aplicó la prueba U de MannWhitney. Además, se clasificaron las mutaciones de acuerdo con su efecto biológico como se describió anteriormente.

Los análisis se realizaron con Stata™, versión 17, y Jamovi™, versión 2.3.28. Las gráficas se realizaron con Python, versión 3.8.1, u otros, dependiendo de la necesidad para cada tipo de análisis.

### Consideraciones éticas

Este estudio se llevó a cabo de acuerdo con los principios establecidos en la declaración de Helsinki. Los pacientes o sus representantes legales otorgaron su consentimiento informado para participar en esta investigación, que involucró una cohorte de pacientes hospitalizados en el área metropolitana del valle de Aburrá, Medellín, entre octubre del 2021 y febrero del 2023.

Las aprobaciones éticas fueron otorgadas según las siguientes actas:

- Clínica Medellín: acta N° CEI-20 del 15 de octubre de 2021,
- San Vicente Fundación: acta N° 30-2021,
- Hospital General de Medellín: acta N° 13-09112021,
- Hospital Pablo Tobón Uribe: acta N° 23/2021,
- Clínica Las Américas: acta N° 178 del 20 de diciembre de 2021,
- IPS Universitaria: acta N° 178,
- Clínica Universitaria Bolivariana: acta N° 02 de 2022 y
- Clínica CES: acta N° 191.

## Resultados

**Figura 2:**
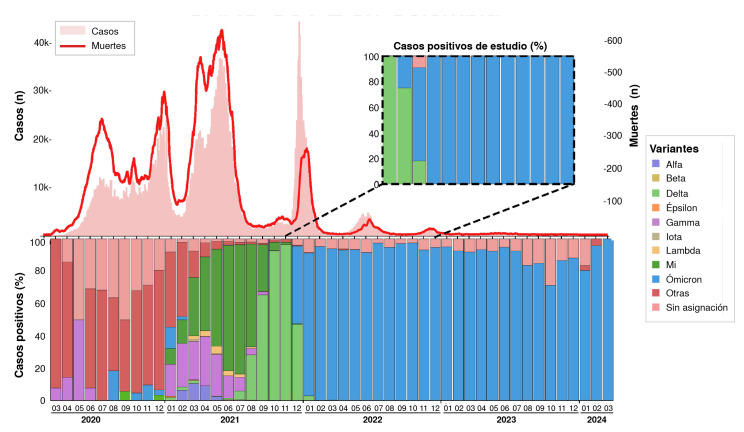
Prevalencia de las variantes de SARS-CoV-2 en Colombia. Prevalencia del SARS-CoV-2, expresada en número de casos por mes y porcentaje de distribución de variantes de este estudio.

Durante el periodo del estudio (octubre del 2021 a febrero del 2023), se incluyeron 131 pacientes. De estos, 63 resultaron con muestras positivas para SARS-CoV-2 y 60 alcanzaron una cobertura mínima del 40 % del genoma completo mediante secuenciación (figura 2). En este grupo, la mediana de edad fue de 69 años (rango intercuartílico: 59 a 78), con el 40 % de pacientes de sexo masculino, y con comorbilidad de alto riesgo para enfermedad grave por SARS-CoV-2. En cuanto al estado inmunológico, el 87 % había recibido, al menos, una dosis de vacuna y el 50 % contaba con una dosis de refuerzo (cuadro 4).

**Cuadro 4:** Características de la cohorte según variante (delta vs. ómicron)

Característica		Total	Delta	Ómicron	p
		(n = 62)*	(n = 9)	(n = 53)	
Demográficas					
	Edad, años [mediana (RIC)]	69 (59-78)	65 (57-69)	70 (60-79)	0,3
	Sexo masculino (%)	25 (40,3)	5 (55,6)	20 (37,7)	0,5
Comorbilidades					
	Hipertensión [n (%)]	39 (62,9)	3 (33,3)	36 (67,9)	0,066
	EPOC [n (%)]	12 (19,7)	2 (22,2)	10 (19,2)	0,9
	Diabetes [n (%)]	19 (30,6)	2 (22,2)	17 (32,1)	0,7
	Obesidad [n (%)]	27 (43,5)	3 (33,3)	24 (45,3)	0,7
	Cáncer [n (%)]	12 (19,4)	2 (22,2)	10 (18,9)	0,9
	Inmunosupresión [n (%)]	30 (48,4)	3 (33,3)	27 (50,9)	0,5
Estado de vacunación e inmunidad					
	Vacunación [n (%)]	54 (87,1)	7 (77,8)	47 (88,7)	0,3
	Primer refuerzo [n (%)]	31 (50,0)	1 (11,1)	30 (56,6)	0,026
	ELISA anti-espícula IgG [n (%)]	49 (79,0)	6 (66,7)	43 (81,1)	0,4
	CMIA_ARCHITECT_anti-espícula IgG [n (%)]	51 (82,3)	6 (66,7)	45 (84,9)	0,2
	Anticuerpos neutralizantes [n (%)]	53 (85,5)	7 (77,8)	46 (86,8)	0,6
Comportamiento durante infección aguda por SARS-CoV-2					
	Clasificación				
	Crítico [n (%)]	26 (41,9)	9 (100,0)	17 (32,1)	<0,001
	Grave [n (%)]	21 (33,9)	0 (0,0)	21 (39,6)	<0,001
	Moderado [n (%)]	15 (24,2)	0 (0,0)	15 (28,3)	<0,001
Ingreso UCI [n (%)]		27 (43,5)	9 (100,0)	18 (34,0)	<0,001
Soporte respiratorio [n (%)]		25 (40,3)	9 (100,0)	16 (30,2)	<0,001
Choque [n (%)]		15 (24,2)	5 (55,6)	10 (18,9)	0,031
Días de hospitalización [mediana (RIC)]		8,5 (4-25)	27 (21-36)	7 (4-19)	0,006
Días en UCI [mediana (RIC)]		0 (0-13)	21 (13-25)	0 (0-10)	<0,001
Mortalidad día 28 [n (%)]		9 (14,5)	3 (33,3)	6 (11,3)	0,11

* Se excluye un paciente clasificado como "Otros".

EPOC: enfermedad pulmonar obstructiva crónica; IgG: Inmunoglobulina G; RIC: rango intercuartiílico; UCI: unidad de cuidados intensivos

**Figura 3:**
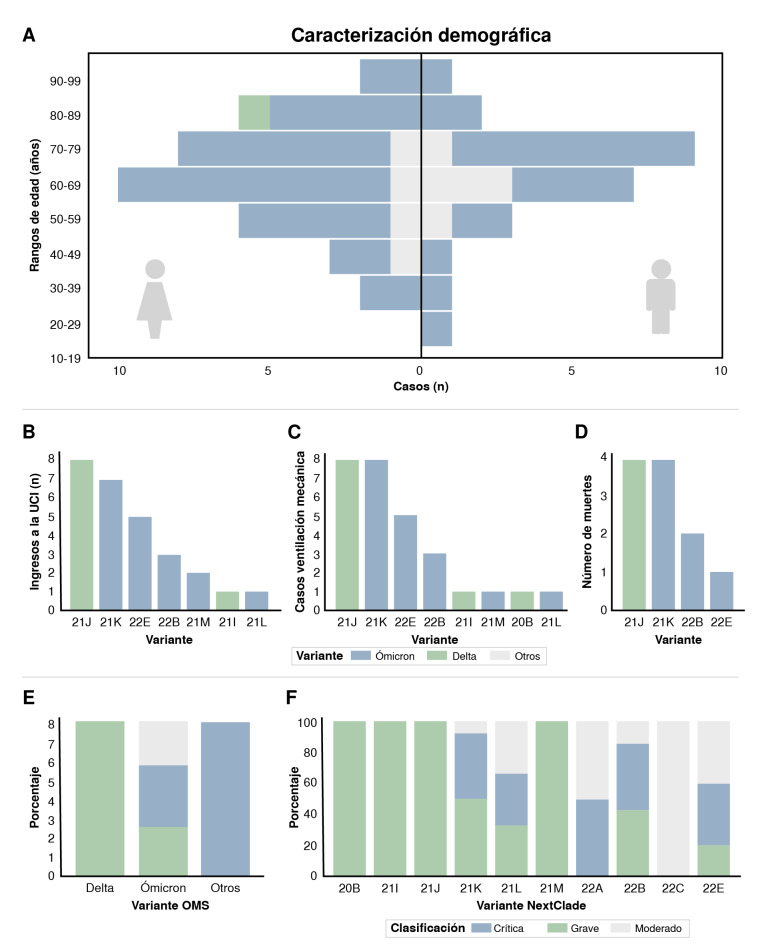
Distribución de variables clínicas por variante

OMS: Organización Mundial de la Salud; SARS-CoV-2: *Severe Acute Respiratory Syndrome Coronavirus 2*.

La mayoría de los casos correspondió a la variante ómicron, con una distribución amplia en todos los grupos etarios. Esta variante fue predominante tanto en hombres como en mujeres, aunque en el caso de las mujeres se concentró en edades más avanzadas. También se identificaron casos asociados a la variante delta, con menor frecuencia y mayor dispersión entre los grupos de edad (figura 3A). Los pacientes con esta variante presentaron un perfil clínico más grave en comparación con los infectados por la variante ómicron (cuadro 4 y figura 3B-F).

A. Caracterización demográfica de los pacientes hospitalizados con infección por SARS-CoV-2, distribuidos por rangos de edad de 10 años. La gráfica muestra el número de casos según sexo: a la derecha, pacientes femeninas; a la izquierda, pacientes masculinos. Cada barra está coloreada según la variante del virus identificada: ómicron (azul), delta (gris) u otros (verde).

B-D. Casos que ingresaron a la unidad de cuidados intensivos, recibieron repiración mecánica asistida y de acuerdo con el estudio, según la asignación de su variante en la nomenclatura Nextclade. Se coloreó la correspondencia de su variante en la nomenclatura de la OMS.

E-F. Gravedad clínica de la enfermedad (crítica, grave y moderada) según la asignación de las variantes en la nomenclatura OMS y Nextclade, respectivamente.

### Análisis filogenético

De las 63 muestras analizadas, 53 correspondieron a la variante ómicron, 9 a la variante delta y 1 a la categoría de "otros", de acuerdo con la nomenclatura de la OMS. El análisis filogenético evidenció una predominancia inicial de la variante delta, la cual se mantuvo hasta comienzos de enero del 2022; a partir de ese momento, todos los casos secuenciados correspondieron a la variante ómicron.

**Figura 4:**
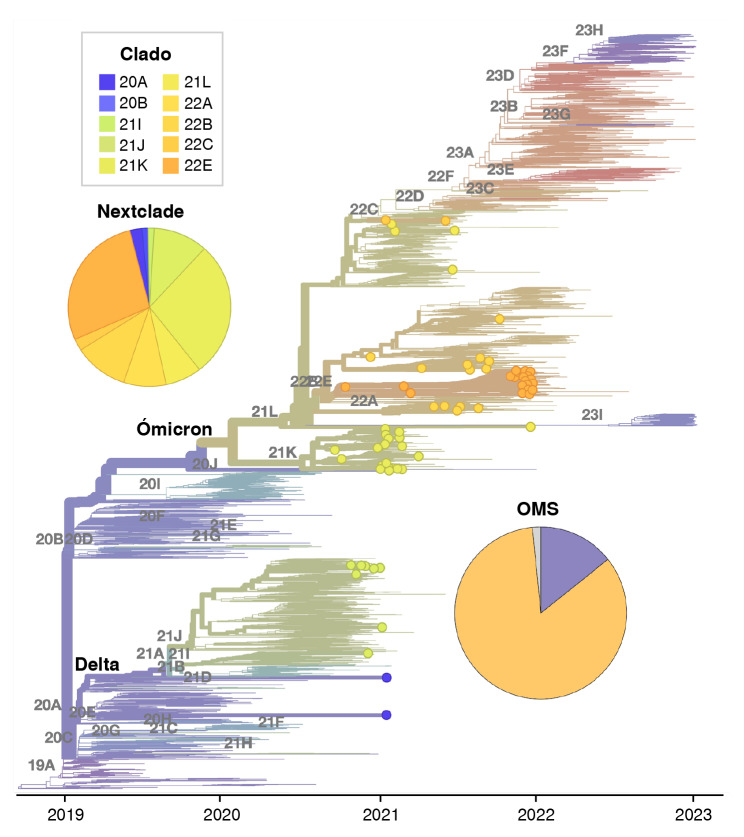
Filogenia de SARS-CoV-2. Análisis filogenético del estudio comparadas con secuencias reportadas a nivel mundial colectadas desde GISAID. Las secuencias del estudio se resaltan con un círculo al final de las ramas.

OMS: Organización Mundial de la Salud; GISAID: *Global Initiative on Sharing All Influenza Data;* SARS-CoV-2: *Severe Acute Respiratory Syndrome* - Coronavirus 2

Para la variante delta, 8 casos se clasificaron como 21J y un caso como 21I. Entre las muestras de ómicron, 20 pertenecían al clado 22E, 13 al 21K, 7 al 22B, 6 al 22A, 2 al 21L, 1 al 21M y 1 al 22C (figura 4). Finalmente, la secuencia clasificada como "otros" se asignó al clado 20B.

### Clasificación clínica de la COVID-19 según análisis filogenético

Al analizar la gravedad de la enfermedad en relación con las variantes estudiadas, se evidenció que la variante delta presentó mayor proporción de enfermedad crítica (figura 3E). El 100 % de los casos de la variantae delta fueron clasificados como críticos. En contraste, para la variante ómicron, solo el 30 % de los casos fueron críticos: el 40 % graves y el 30 % moderados.

En el análisis detallado por clados, utilizando la clasificación de Nextclade (figura 3F), se observó que todos los casos de los clados 21J y 21I correspondientes a la variante delta se clasificaron como críticos. La variante 21M, perteneciente a la variante ómicron, fue la única que presentó todos sus casos como críticos, mientras que las demás variantes de ómicron se clasificaron como graves o moderadas.

Al evaluar las variantes en relación con las variables clínicas -como el ingreso a la unidad de cuidados intensivos, respiración mecánica asistida y mortalidad (figuras 3B-C)-, se encontró que el clado 21J de la variante delta tuvo el mayor número de ingresos a la unidad de cuidados intesivos. Esta misma variante se identificó con mayor frecuencia en los casos que requirieron respiración mecánica asistida o que fallecieron, seguida por la variante 21K de ómicron, que se situó en el segundo lugar en cuanto al número de casos que ingresaron a la unidad de cuidados intensivos.

### Mutaciones en la proteína espícula del SARS-CoV-2

**Figura 5:**
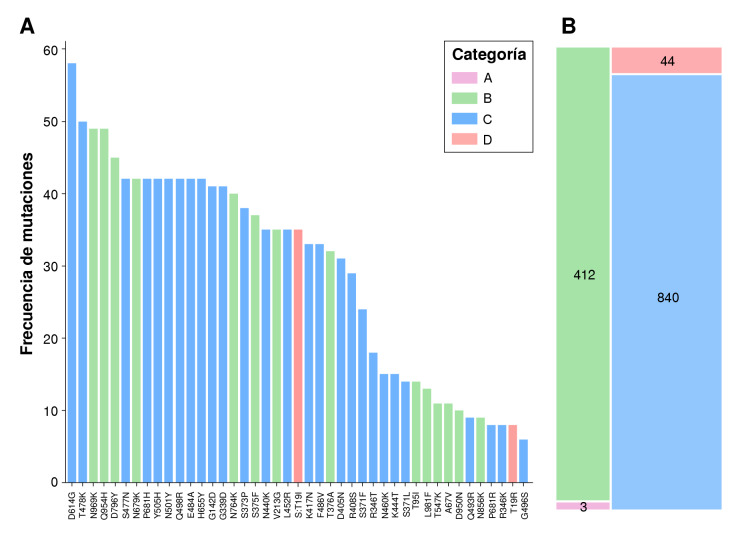
Clasificación de mutaciones por categoría en la proteína espícula. **A.** Frecuencia de las 50 mutaciones más frecuentes. **B.** Número de mutaciones que pertenece a cada categoría.

Como se ilustra en la figura 5A, se identificaron mutaciones en las cuatro categorías definidas en el cuadro 2, de acuerdo con su efecto biológico. De un total de 1299, la mayoría (64,6 %, n = 840) fueron clasificadas en la categoría C, seguidas por la categoría B (31,7 %, n = 412); mientras que las mutaciones de la categoría D (8,7 %, n = 114) y A (0,2 %, n = 3) fueron la minoritarias.

Asimismo, en la figura 5B se observa que la mutación D614G fue la más común dentro de la categoría C, presente en el 96 % de los virus estudiados; similar a lo notificado en CovSurver, donde aparece en el 99,3 % de los virus reportados.

En la categoría B, las mutaciones N969K y Q954H fueron las más frecuentes (49 de 412 cada una), presentes en el 81 % de las secuencias de este estudio, mientras que en GISAID se reportan en el 78,6 y en el 78,7 % de las cepas, respectivamente. Ambas mutaciones fueron excluidas de las cepas de la variante ómicron y se encuentran localizadas en la región de repetición heptádica 1 de la subunidad S2 de la proteína espícula.

En la categoría D solo se identificaron dos mutaciones, y la T19I fue la más frecuente (35 de 44), correspondiente al 58,3 % de las cepas estudiadas, mientras que en CovSurver se presentó en el 32,3 % de las cepas registradas en el servidor.

**Figura 6:**
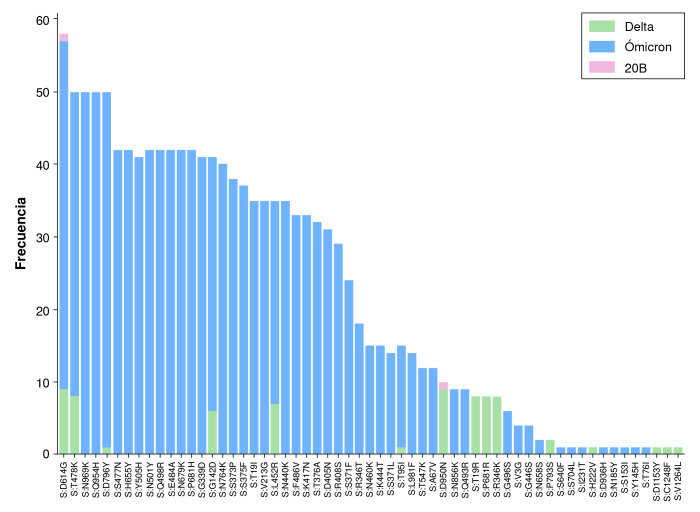
Sustituciones por variante en proteína espicula. Frecuencia de sustitución de aminoácidos por variante delta, ómicron y 20B.

En el cuadro 3 y figura 6 se presenta información adicional de las mutaciones que se encuentran, al menos, en el 25 % de las secuencias de estudio.

### Mutaciones del gen S del virus SARS-CoV-2

**Figura 7:**
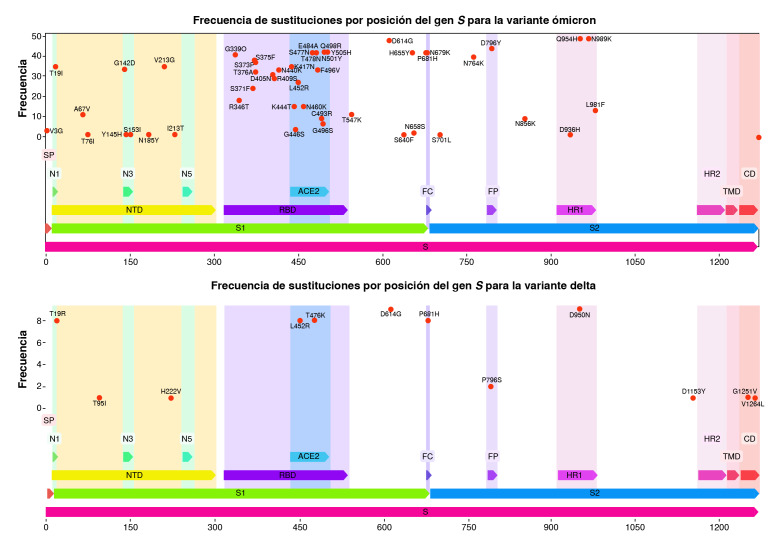
Mutaciones de SARS-CoV-2 en el gen de la proteína espícula. La gráfica muestra la frecuencia y la posición donde se encontraron cambios de aminoácidos a lo largo del gen S para la variante delta (A) y ómicron (B).

El gen S es el responsable de codificar la proteína espícula y se divide en dos subunidades principales: S1 y S2. La subunidad S1 contiene el dominio de unión al receptor, que es responsable de reconocer y unirse al receptor de la enzima convertidora de angiotensina 2 en las células humanas. Entre las mutaciones más destacadas que encontramos en esta región están: S371F, K417N, D405N, R408S, N440K, S477N y F486V, G339D, S373P, S375F, L452R, T478K, Q498R y N501Y (figuras 7 y 8).

SARS-CoV-2: *Severe Acute Respiratory Syndrome Coronavirus 2.*

**Figura 8:**
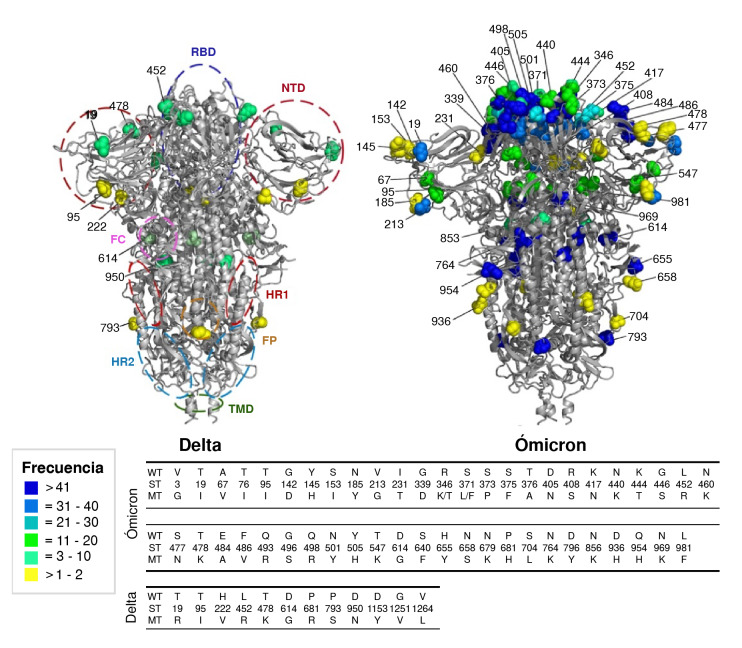
Mutaciones de SARS-CoV-2 en la estructura de la proteína espicula. Las mutaciones presentes en el estudio se ubicaron en la estructura de la proteína espicula para las variantes delta y ómicron. Los colores fueron asignados segín la frecuencia de las mutaciones en el estudio. La tabla enumera la posición en la que se da el cambio y el aminoácido desde la secuencia de referencia hasta la mutante. Las regiones de RBD y NTD se resaltan en azul y rojo correspondientemente.

RBD: *Receptor binding domain* (dominio de unión al ligando); NTD: *N-terminal domain* (dominio N-terminal); SARS-CoV-2: *Severe Acute Respiratory Syndrome Coronavirus 2.*

Además del dominio de unión al receptor, la subunidad S1 también incluye el dominio N-terminal, que contribuye a la unión a los receptores celulares. En esta región se observaron mutaciones como T19I y G142D.

La subunidad S2 contiene elementos críticos para la fusión de membranas, facilitando la entrada del material genético viral a la célula huésped mediante cambios conformacionales. En esta región se encuentra la repetición heptádica 1, donde están las mutaciones Q954H y N969K, el motivo de repetición heptádico 2, la región de inserción transmembrana y el péptido de fusión, el cual presenta la mutación N769Y.

Finalmente, se identificaron mutaciones en el sitio de escisión de la furina, que separa las subunidades S1 y S2. La variante ómicron presentó la mutación P681H, mientras que la variante delta reportó la mutación P651R.

## Discusión

La aparición y propagación de variantes del SARS-CoV-2 fue motivo de gran preocupación durante la pandemia de la COVID-19. Durante el desarrollo del estudio, Colombia ya había experimentado tres grandes olas epidémicas (agosto del 2020, enero del 2021 y junio del 2021) y, en ese momento, se implementaban activamente campañas de vacunación con dosis de refuerzo ^10^.

En nuestro estudio, basado en las secuencias obtenidas y analizadas entre el 2021 y el 2023, el análisis filogenético evidenció una predominancia inicial de la variante delta, la cual fue posteriormente reemplazada por la variante ómicron, que se mantuvo hasta el final del periodo de análisis. Este patrón coincide con la tendencia general de las variantes en Colombia.

Las variantes previas a ómicron, como delta y mi, tuvieron un impacto significativo en el panorama epidemiológico del país, con distintos picos en el número de casos y en las cifras de mortalidad. Cabe destacar que la variante mi fue catalogada como "de alto interés" por la OMS, debido a su alto potencial para evadir la respuesta inmunológica de los pacientes con infecciones previas o ya vacunados ^32^.

A mediados del 2021, la variante delta, detectada originalmente en India, comenzó a reemplazar a la variante mi como la cepa dominante en Colombia. La variante delta presentó una mayor transmisibilidad y capacidad para causar enfermedad más grave, lo que contribuyó al rápido desplazamiento de la variante mi y de otras variantes circulantes ^33^^,^^34^. A finales del año 2021, emergió la variante ómicron, que rápidamente superó en número de casos a la variante delta debido a su mayor transmisibilidad, convirtiéndose en la variante dominante tanto en Colombia como en el resto del mundo ^35^.

Nuestros resultados, que evalúan la relación entre las variantes del SARS-CoV-2 y las variables clínicas (ingreso a la unidad de cuidados intensivos, respiración mecánica asistida y mortalidad), evidencian que la variante delta tuvo un mayor impacto clínico en comparación con la ómicron. Según la literatura, la variante delta es más mortal y altamente transmisible, generando signos y síntomas de enfermedad grave, recuperación lenta y mayores tasas de hospitalización ^16^^,^^17^^,^^36^^-^^39^. Cabe resaltar que la cohorte analizada corresponde a una población adulta con factores de riesgo para la enfermedad grave por SARS-CoV-2 y que, en su mayoría, estaban vacunados (algunos contaban con dosis de refuerzo conforme avanzaba el Plan Nacional de Vacunación).

Estos hallazgos subrayan la gravedad de las infecciones, incluso en personas vacunadas que requirieron hospitalización, y resaltan la importancia de la vigilancia genómica como herramienta esencial para el monitoreo de la evolución viral y la evaluación de sus posibles efectos biológicos ^20^.

La proteína espícula juega un papel fundamental para la entrada del virus a las células humanas al unirse al receptor enzima convertidora de angiotensina 2 ^40^. Por tanto, algunas mutaciones de dicha proteína pueden aumentar la transmisibilidad del virus, facilitar la evasión de la respuesta inmunitaria, alterar la eficacia de las vacunas e incrementar la gravedad de la enfermedad.

Entre las mutaciones identificadas, la D614G, que consiste en la sustitución del ácido aspártico por glicina en el aminoácido 614, fue la más frecuente de la categoría C ^41^. Esta mutación confiere al virus habilidades infecciosas significativamente mayores, con un aumento entre 2,6 y 9,3 veces en varias líneas celulares ^42^^.^

En los individuos infectados, la mutación D614G se asocia con ciclos de reacción en cadena de la polimerasa con transcriptasa inversa más bajos, lo que indica una carga viral más alta en las vías respiratorias superiores. Sin embargo, esta mutación no está asociada con una mayor seriedad de la enfermedad ^43^.

Las mutaciones N969K y Q954H fueron las más frecuentes en la categoría B. Según Pastorio *et al.*^44^, estas mutaciones reducen la eficiencia de la infección mediada por la proteína S, al disminuir la eficiencia de fusión del virus con la célula huésped.

En la categoría D, se identificaron dos mutaciones, y la T19I era la más frecuente. Esta mutación elimina un posible sitio de glicosilación, lo que afecta las propiedades antigénicas de la proteína espícula, contribuyendo potencialmente a cambios en la respuesta inmunitaria de huésped ^45^.

Entre las mutaciones de las secuencias estudiadas a lo largo del gen *S,* se encontraron varias relacionadas con la evasión de la respuesta inmunitaria y con alteraciones antigénicas, como S371F, K417N, D405N, R408S, N440K, S477N y F486V. Estas mutaciones inducen cambios conformacionales (S371F y R408S) y electrostáticos (D405N y K417N), entre otros, que permiten al virus evadir la respuesta inmunológica del huésped ^46^^-^^50^.

Otras mutaciones, como G339D, S373P, S375F, L452R, T478K, Q498R y N501Y, están relacionadas con un aumento en la afinidad por el receptor enzima convertidora de angiotensina 2 ^51^^-^^54^, lo que potencialmente mejora la eficiencia del ingreso del virus a la célula y su capacidad infecciosa.

En el dominio N-terminal, contenido en la subunidad S1, se destacaron mutaciones como T19I y G142D, las cuales facilitan el escape de los anticuerpos neutralizantes ^45^^,^^50^^,^^55^. La subunidad S2 contiene elementos críticos para la fusión de membranas, lo cual permite que las mutaciones en esta región induzcan cambios conformacionales que faciliten la entrada del material genético viral a la célula huésped ^56^. Esta región incluye la repetición heptádica 1, que presenta las mutaciones Q954H y N969K. También está involucrada la repetición heptádica 2, la región de inserción transmembrana y péptido de fusión, donde se identificó la mutación N769Y, la cual sugiere una reducción en el potencial de neutralización por anticuerpos dirigidos al dominio S1 ^57^^,^^58^.

Finalmente, se encontraron mutaciones en el sitio de escisión de la furina, el cual separa los dominios S1 y S2 ^59^.

La variante ómicron presentó la mutación P681H, que mejora la escisión de la proteína S1-S2, y que promueve una mayor entrada a la célula. En contraste, la variante delta reportó la mutación P651R, asociada con una mayor infección célula a célula y con un aumento en la formación de sincitios que evaden la neutralización por anticuerpos. Esto podría ayudar a explicar la mayor patogénesis y propagación de la variante delta ^60^^,^^61^.

La principal fortaleza de este estudio radica en el análisis detallado de las mutaciones virales y sus posibles efectos biológicos, realizado de forma longitudinal a lo largo de la evolución de la pandemia.

Como limitación, durante el periodo de estudio, la muestra diagnóstica inicial se destinaba a la vigilancia genómica nacional. Por esta razón, muchas de las muestras tomadas dentro de las primeras 72 horas resultaron negativas o con cobertura insuficiente para secuenciación, lo que redujo el número de muestras analizadas. Para los análisis se incluyeron las muestras con una cobertura superior al 40 %, siempre que contaran con confirmación de la variante mediante la prueba de reacción en cadena de la polimerasa en tiempo real con transcripción inversa específica dirigida a variantes de interés.

En resumen, la variante ómicron fue la predominante en las secuencias analizadas en nuestro estudio. Los casos correspondientes a la variante delta estuvieron relacionados con mayor gravedad clínica de la enfermedad. Se identificaron mutaciones de la proteína espícula en las cuatro categorías (A, B, C, D), así como mutaciones específicas del gen *S.*

En conclusión, la vigilancia genómica continúa siendo una herramienta esencial para el monitoreo de virus emergentes como el SARS-CoV-2. Su implementación permite una respuesta oportuna frente a la evolución viral y contribuye de manera significativa al fortalecimiento de los sistemas de salud pública, especialmente en contextos de alta transmisibilidad y aparición de nuevas variantes.

## Archivos suplementarios

Anexo 1. Formulario de encuesta clínico-epidemiológica

**Encuesta clínica:** a cada participante se le aplicó un formulario de encuesta clínico-epidemiológica para registrar información demográfica y médica completa. Este incluyó el historial de uso de medicamento, antecedentes de infecciones previas por la COVID-19 y vacunación. También se documentó la progresión de síntomas actuales y condiciones médicas preexistentes, como hipertensión, diabetes mellitus, asma, enfermedad pulmonar obstructiva crónica, enfermedad de las arterias coronarias, tuberculosis, enfermedad renal y obesidad. Además, se recopiló información sobre antecedentes de inmunosupresión, incluyendo tratamientos previos con quimioterapia, terapias biológicas y enfermedades autoinmunes. Finalmente, se incluyeron datos socioeconómicos y culturales para una caracterización integral de los participantes.

Encuesta paciente hospitalizado: se implementó para evaluar la fase aguda de la infección por SARS-CoV-2 en pacientes hospitalizados, registrando la duración de la estancia, traslados a unidades de cuidados intensivos (UCI) o especiales (UCE) y la clasificación de la gravedad de la enfermedad. Se realizaron evaluaciones clínicas periódicas y se verificaron las fechas de egreso, con el objetivo de registrar el estado vital al alta y la mortalidad a los 28 días posteriores a la infección. Adicionalmente, se hizo seguimiento de los días sin requerimiento de ventilación mecánica, diálisis o tratamiento por choque. Para la evaluación a los 28 días, se contactó a los participantes por vía telefónica y se diligenció el formulario correspondiente.

## References

[1] Organización Mundial de la Salud Información básica sobre la COVID-19.

[2] Duffy S (2018). Why are RNA virus mutation rates so damn high?. PLOS Biol.

[3] Robson F, Khan KS, Le TK, Paris C, Demirbag S, Barfuss P (2020). Coronavirus RNA proofreading: molecular basis and therapeutic targeting. Mol Cell.

[4] Helmy YA, Fawzy M, Elaswad A, Sobieh A, Kenney SP, Shehata AA (2020). The COVID-19 pandemic: a comprehensive review of taxonomy, genetics, epidemiology, diagnosis, treatment, and control. J Clin Med.

[5] Markov PV, Ghafari M, Beer M, Lythgoe K, Simmonds P, Stilianakis NI (2023). The evolution of SARS-CoV-2. Nat Rev Microbiol.

[6] Organización Mundial de la Salud Declaración sobre la actualización de las definiciones de trabajo y del sistema de seguimiento de las variantes preocupantes y las variantes de interés del SARS-CoV-2.

[7] O’Toole Á, Pybus OG, Abram ME, Kelly EJ, Rambaut A (2022). Pango lineage designation and assignment using SARS-CoV-2 spike gene nucleotide sequences. BMC Genomics.

[8] Bedford T, Hodcroft E, Neher R Updated Nextstrain SARS-CoV-2 clade naming strategy.

[9] Viana R, Moyo S, Amoako DG, Tegally H, Scheepers C, Althaus CL (2022). Rapid epidemic expansion of the SARS-CoV-2 Omicron variant in southern Africa. Nature.

[10] Prada SI, García-García MP, Guzmán J (2022). COVID-19 response in Colombia: Hits and misses. Health Policy Technol.

[11] Hernández-Ortiz J, Cardona A, Ciuoderis K, Averhoff F, Maya MA, Cloherty G (2022). Assessment of SARS-CoV-2 mu variant emergence and spread in Colombia. JAMA Netw Open.

[12] Álvarez-Díaz DA, Ruiz-Moreno HA, Zapata-Bedoya S, Franco-Muñoz C, Laiton-Donato K, Ferro C (2022). Clinical outcomes associated with mu variant infection during the third epidemic peak of COVID-19 in Colombia. Int J Infect Dis.

[13] Patiño LH, Ballesteros N, Muñoz M, Ramírez AL, Luna N, Castañeda S (2023). Mu SARSCoV- 2 (B.1.621) variant: a genomic snapshot across the Colombian-Venezuelan border. J Med Virol.

[14] Laiton-Donato K, Franco-Muñoz C, Álvarez-Díaz DA, Ruiz-Moreno HA, Usme-Ciro JA, Prada DA (2021). Characterization of the emerging B.1.621 variant of interest of SARSCoV-2. Infect Genet Evol.

[15] Jiménez-Silva C, Rivero R, Douglas J, Bouckaert R, Villabona-Arenas CJ, Atkins KE (2023). Genomic epidemiology of SARS-CoV-2 variants during the first two years of the pandemic in Colombia. Commun Med.

[16] Varea-Jiménez E, Aznar E, Vega-Piris L, Martínez EV, Mazagatos C, García L (2024). Comparative severity of COVID-19 cases caused by alpha, delta or omicron SARS-CoV-2 variants and its association with vaccination. Enferm Infecc Microbiol Clin.

[17] Nyberg T, Ferguson NM, Nash SG, Webster HH, Flaxman S, Andrews N (2022). Comparative analysis of the risks of hospitalisation and death associated with SARS-CoV-2 omicron (B.1.1.529) and delta (B.1.617.2) variants in England: a cohort study. Lancet.

[18] Cojocaru C, Cojocaru E, Turcanu AM, Zaharia DC (2022). Clinical challenges of SARS-CoV-2 variants. Exp Ther Med.

[19] Hacisuleyman E, Hale C, Saito Y, Blachere NE, Bergh M, Conlon E (2021). Vaccine breakthrough infections with SARS-CoV-2 variants. N Engl J Med.

[20] Hernández-Ortiz OH, Naranjo AF, Cadavid JJV, Echavez G, Moreno-Bedoya S, Gil B (2025). Risk factors for breakthrough acute SARS-CoV-2 infections in fully vaccinated individuals: a case-control study nested in a prospective cohort in Medellín, Colombia. J Med Virol.

[21] Corman VM, Landt O, Kaiser M, Molenkamp R, Meijer A, Chu DK (2020). Detection of 2019 novel coronavirus (2019-nCoV) by real-time RT-PCR. Euro Surveill.

[22] Artic-network GitHub. artic-ncov2019/primer_schemes at master artic-network/articncov2019.

[23] GitHub Proposal for lineage within B.1 with N501Y, E484K, R346K, P681H Spike substitutions Issue #57 cov-lineages/pango-designation.

[24] Jolly B, Scaria V. (2021). Computational analysis and phylogenetic clustering of SARS-CoV-2 genomes. Bio-Protoc.

[25] Hadfield J, Megill C, Bell SM, Huddleston J, Potter B, Callender C (2018). Nextstrain: realtime tracking of pathogen evolution. Bioinformatics.

[26] Trifinopoulos J, Nguyen LT, von Haeseler A, Minh BQ (2016). W-IQ-TREE: a fast online phylogenetic tool for maximum likelihood analysis. Nucleic Acids Res.

[27] Katoh K, Standley DM (2013). MAFFT multiple sequence alignment software version 7: improvements in performance and usability. Mol Biol Evol.

[28] Larsson A (2014). AliView: a fast and lightweight alignment viewer and editor for large datasets. Bioinformatics.

[29] Zulkower V, Rosser S (2020). DNA features viewer: a sequence annotation formatting and plotting library for Python. Bioinformatics.

[30] Re3data.Org (2012). GISAID. re3data.org - Registry of Research Data Repositories.

[31] Zhang C, Wang Y, Zhu Y, Liu C, Gu C, Xu S (2021). Development and structural basis of a two-MAb cocktail for treating SARS-CoV-2 infections. Nat Commun.

[32] Uriu K, Kimura I, Shirakawa K, Takaori-Kondo A, Nakada TA, Kaneda A (2021). Neutralization of the SARS-CoV-2 Mu variant by convalescent and vaccine serum. N Engl J Med.

[33] Orf GS, Pérez LJ, Ciuoderis K, Cardona A, Villegas S, Hernández-Ortiz JP (2023). The Principles of SARS-CoV-2 intervariant competition are exemplified in the pre-omicron era of the Colombian Epidemic. Microbiol Spectr.

[34] Xie X, Han JB, Ma G, Feng XL, Li X, Zou QC (2021). Emerging SARS-CoV-2 B.1.621/ Mu variant is prominently resistant to inactivated vaccine-elicited antibodies. Zool Res.

[35] Chrysostomou AC, Vrancken B, Haralambous C, Alexandrou M, Gregoriou I, Ioannides M (2023). Unraveling the dynamics of Omicron (BA.1, BA.2, and BA.5) waves and emergence of the deltacton variant: genomic epidemiology of the SARS-CoV-2 epidemic in Cyprus (Oct 2021-Oct 2022). Viruses.

[36] Anwar MZ, Lodhi MS, Khan MT, Khan MI, Sharif S (2022). Coronavirus genomes and unique mutations in structural and non-structural proteins in Pakistani SARS-CoV-2 delta variants during the fourth wave of the pandemic. Genes.

[37] Twohig KA, Nyberg T, Zaidi A, Thelwall S, Sinnathamby MA, Aliabadi S (2022). Hospital admission and emergency care attendance risk for SARS-CoV-2 delta (B.1.617.2) compared with alpha (B.1.1.7) variants of concern: a cohort study. Lancet Infect Dis.

[38] Veneti L, Bøås H, Bråthen Kristoffersen A, Stålcrantz J, Bragstad K, Hungnes O (2022). Reduced risk of hospitalisation among reported COVID-19 cases infected with the SARSCoV-2 Omicron BA.1 variant compared with the Delta variant, Norway, December 2021 to January 2022. Euro Surveill.

[39] Kumar N, Quadri S, AlAwadhi AI, AlQahtani M (2022). COVID-19 recovery patterns across alpha (B.1.1.7) and Delta (B.1.617.2) Variants of SARS-CoV-2. Front Immunol.

[40] Huang Y, Yang C, Xu XF, Xu W, Liu SW (2020). Structural and functional properties of SARS-CoV-2 spike protein: potential antivirus drug development for COVID-19. Acta Pharmacol Sin.

[41] Bhattacharya M, Chatterjee S, Sharma AR, Agoramoorthy G, Chakraborty C (2021). D614G mutation and SARS-CoV-2: impact on S-protein structure, function, infectivity, and immunity. Appl Microbiol Biotechnol.

[42] Thakur V, Bhola S, Thakur P, Patel SKS, Kulshrestha S, Ratho RK (2022). Waves and variants of SARS-CoV-2: understanding the causes and effect of the COVID-19 catastrophe. Infection.

[43] Korber B, Fischer WM, Gnanakaran S, Yoon H, Theiler J, Abfalterer W (2020). Tracking changes in SARS-CoV-2 spike: Evidence that D614G increases infectivity of the COVID-19 virus. Cell.

[44] Pastorio C, Zech F, Noettger S, Jung C, Jacob T, Sanderson T (2022). Determinants of Spike infectivity, processing, and neutralization in SARS-CoV-2 Omicron subvariants BA.1 and BA.2. Cell Host Microbe.

[45] Van Poelvoorde LAE, Picalausa C, Gobbo A, Verhaegen B, Lesenfants M, Herman P (2023). Development of a droplet digital PCR to monitor SARS-CoV-2 Omicron variant BA.2 in wastewater samples. Microorganisms.

[46] Bugatti A, Filippini F, Messali S, Giovanetti M, Ravelli C, Zani A (2023). The D405N mutation in the Spike protein of SARS-CoV-2 Omicron BA.5 inhibits spike/integrins interaction and viral infection of human lung microvascular endothelial cells. Viruses.

[47] Cao Y, Wang J, Jian F, Xiao T, Song W, Yisimayi A (2022). Omicron escapes the majority of existing SARS-CoV-2 neutralizing antibodies. Nature.

[48] Kullappan M, Mary U, Ambrose JM, Veeraraghavan VP, Surapaneni KM (2023). Elucidating the role of N440K mutation in SARS-CoV-2 spike - ACE-2 binding affinity and COVID-19 severity by virtual screening, molecular docking and dynamics approach. J Biomol Struct Dyn.

[49] Moriyama S, Anraku Y, Taminishi S, Adachi Y, Kuroda D, Kita S (2023). Structural delineation and computational design of SARS-CoV-2-neutralizing antibodies against Omicron subvariants. Nat Commun.

[50] Wang L, Møhlenberg M, Wang P, Zhou H (2023). Immune evasion of neutralizing antibodies by SARS-CoV-2 Omicron. Cytokine Growth Factor Rev.

[51] Deng X, Garcia-Knight MA, Khalid MM, Servellita V, Wang C, Morris MK (2021). Transmission, infectivity, and neutralization of a spike L452R SARS-CoV-2 variant. Cell.

[52] Di Giacomo S, Mercatelli D, Rakhimov A, Giorgi FM (2021). Preliminary report on severe acute respiratory syndrome coronavirus 2 (SARS-CoV-2) Spike mutation T478K. J Med Virol.

[53] Starr TN, Greaney AJ, Hilton SK, Ellis D, Crawford KHD, Dingens AS (2020). Deep mutational scanning of SARS-CoV-2 receptor binding domain reveals constraints on folding and ACE2 binding. Cell.

[54] Kumar S, Thambiraja TS, Karuppanan K, Subramaniam G (2022). Omicron and Delta variant of SARS-CoV-2: A comparative computational study of spike protein. J Med Virol.

[55] Wang Q, Ye SB, Zhou ZJ, Song AL, Zhu X, Peng JM (2023). Key mutations in the spike protein of SARS-CoV-2 affecting neutralization resistance and viral internalization. J Med Virol.

[56] Kumar S, Delipan R, Chakraborty D, Kanjo K, Singh R, Singh N (2023). Mutations in S2 subunit of SARS-CoV-2 Omicron spike strongly influence its conformation, fusogenicity, and neutralization sensitivity. J Virol.

[57] Almalki SSR, Izhari MA, Alyahyawi HE, Alatawi SK, Klufah F, Ahmed WAM (2023). Mutational analysis of circulating Omicron SARS-CoV-2 lineages in the Al-Baha Region of Saudi Arabia. J Multidiscip Healthc.

[58] Yang K, Wang C, Kreutzberger AJB, White KI, Pfuetzner RA, Esquivies L (2023). Structurebased design of a SARS-CoV-2 Omicron-specific inhibitor. Proc Natl Acad Sci USA.

[59] Walls AC, Park YJ, Tortorici MA, Wall A, McGuire AT, Veesler D (2020). Structure, function, and antigenicity of the SARS-CoV-2 Spike glycoprotein. Cell.

[60] Lista MJ, Winstone H, Wilson HD, Dyer A, Pickering S, Galao RP (2022). The P681H mutation in the Spike glycoprotein of the Alpha variant of SARS-CoV-2 escapes IFITM restriction and is necessary for type I interferon resistance. J Virol.

[61] Lubinski B, Fernandes MHV, Frazier L, Tang T, Daniel S, Diel DG (2022). Functional evaluation of the P681H mutation on the proteolytic activation of the SARS-CoV-2 variant B.1.1.7 (Alpha) spike. iScience.

